# Self-Reported Outdoor Activities and Perceived Stress During the First COVID-19 Wave: A Cross-Sectional Study in Poland and Italy

**DOI:** 10.3390/medicina62081564

**Published:** 2026-08-14

**Authors:** Jakub Grabowski, Joanna Stępień, Przemysław M. Waszak, Tomasz Michalski, Roberta Meloni, Maja Grabkowska, Aleksandra Macul-Sanewska, Jakub Rojek, Liliana Lorettu, Iwona Sagan, Aleksandra Brzozowska, Leszek Bidzan

**Affiliations:** 1 Department of Developmental Psychiatry, Psychotic and Geriatric Disorders, Medical University of Gdańsk, 80-282 Gdańsk, Poland; 2 Division of Political Geography, Institute of Socio-Economic Geography and Spatial Management, University of Gdańsk, 80-309 Gdańsk, Poland; 3 Department of Hygiene and Epidemiology, Medical University of Gdańsk, 80-210 Gdańsk, Poland; 4 University of Sassari, 07100 Sassari, Italy; 5 Adult Psychiatry Scientific Circle, Department of Developmental Psychiatry, Psychotic and Geriatric Disorders, Medical University of Gdańsk, 80-282 Gdańsk, Poland; 6 Psychiatric Clinic, Department of Medical, Surgical and Experimental Sciences, University of Sassari, 07100 Sassari, Italy

**Keywords:** public mental health, perceived stress, outdoor physical activity, outdoor sports, prevention, COVID-19 pandemic, lockdown, mental health

## Abstract

*Background and Objectives*: The COVID-19 pandemic and the accompanying containment measures substantially altered everyday life and created conditions that could intensify psychological distress. Physical activity is widely recognized as a factor supporting mental well-being, yet less is known about whether specific forms of outdoor activity were associated with perceived stress during the first pandemic wave. This study aimed to assess the relationship between selected forms of outdoor physical activity and perceived stress during the COVID-19 pandemic among residents of Poland and Italy. *Materials and Methods*: This cross-sectional analysis was based on data collected within a previously published multinational survey conducted during the first wave of the COVID-19 pandemic. The study included respondents from Poland and Italy and examined PSS scores in relation to three broad self-reported agreement items concerning walking in public places, walking in the neighborhood, and outdoor sports such as running or cycling. Responses were analyzed in their original categories and were additionally dichotomized as agreement versus disagreement. Non-parametric tests were used for unadjusted comparisons, and multivariable logistic regression adjusted for country and sociodemographic and health-related factors. *Results*: In unadjusted analyses, respondents who agreed that they walked in public places or engaged in outdoor sports had lower mean PSS scores than those who disagreed (20.40 vs. 21.61 and 20.59 vs. 21.70, respectively; both *p* < 0.001). Respondents who agreed that they walked in their neighborhood had a slightly higher mean PSS score than those who disagreed (21.76 vs. 21.26; *p* = 0.018). After correction of the dichotomized activity variables, none of the three outdoor activity measures were independently associated with high perceived stress in the adjusted model. *Conclusions*: Self-reported walking in public places and outdoor sports were associated with lower PSS scores in unadjusted comparisons, whereas neighborhood walking showed a small association in the opposite direction. None of the three broad activity measures were independently associated with high perceived stress after adjustment. Because the study was cross-sectional and outdoor activity was assessed using simple agreement statements rather than frequency, duration, or intensity measures, these findings should be interpreted cautiously and do not support causal conclusions.

## 1. Introduction

The coronavirus disease 2019 (COVID-19) pandemic represented an unprecedented global public health crisis and was accompanied by extensive restrictions affecting mobility, social contact, work, and daily routines. During the first wave of the COVID-19 pandemic, populations across many countries were exposed to multiple concurrent stressors, including prolonged uncertainty, fear of infection, social isolation, disrupted daily routines, and financial concerns. These pandemic-related stressors were associated with elevated levels of psychological distress, anxiety, depressive symptoms, insomnia, and post-traumatic stress symptoms [1,2,3]. Earlier analyses conducted within the same Polish–Italian survey project demonstrated that perceived stress during the first phase of the pandemic was associated with demographic, socioeconomic, health-related, and behavioral factors, highlighting the broad mental health impact of the crisis [4].

Physical activity (PA) is an established determinant of physical and mental health. Regular exercise has been associated with lower levels of stress, anxiety, and depressive symptoms, as well as with better sleep and overall well-being [5,6,7,8]. During the COVID-19 pandemic, however, opportunities for PA were reduced due to stay-at-home orders, closure of gyms and recreational facilities, and restrictions on public movement. As a result, many individuals reduced their PA levels and increased sedentary time during the pandemic [9,10,11]. At the same time, maintaining PA was recommended as an important strategy to support health and well-being during quarantine periods [12,13].

Several studies conducted during the pandemic suggested that higher levels of PA were associated with lower perceived stress [14,15,16,17,18,19,20,21]. However, much of this evidence conceptualized PA primarily in quantitative terms, focusing on total activity volume, intensity, sedentary time, or MET-based indicators, rather than on the specific behavioral and environmental context in which activity was performed. Consequently, less is known about whether different outdoor behaviors during lockdown conditions were associated with perceived stress in the same way. Walking in the neighborhood, recreational walking in public places, and outdoor sports such as running or cycling may represent distinct forms of activity, differing in accessibility, intensity, regularity, and psychological meaning [22,23].

The present study extends previous work from the same survey project by examining whether selected forms of outdoor PA were associated with perceived stress among respondents from Poland and Italy during the first wave of the COVID-19 pandemic. The Polish–Italian comparison is substantively relevant because the parent analysis showed marked cross-country differences in perceived stress and containment-measure compliance, with Polish respondents reporting higher mean PSS-10 scores than Italian respondents (22.14 vs. 17.01; *p* < 0.01) [4]. Therefore, the two national subsamples should not be treated as merely interchangeable sources of additional observations. Specifically, we assessed walking in the neighborhood, walking in public places, and outdoor sports activity as separate behavioral exposures. We hypothesized that engagement in outdoor PA would be associated with lower perceived stress, with the strongest association expected for outdoor sports activity.

## 2. Materials and Methods

The full sample comprised 7113 respondents, including 6169 participants from Poland (86.7%) and 944 (13.3%) from Italy. The dataset came from a previously published Polish–Italian survey project; detailed information on the recruitment procedure, survey setting, and full demographic context has been reported elsewhere [4]. Briefly, the questionnaire was disseminated through national and local media, regional and municipal websites, social media platforms, and university newsletters to obtain responses from across both countries. The recruitment strategy also partly used virtual snowball sampling. Before taking part in the survey, participants provided electronic informed consent and could withdraw from the study at any stage. All procedures involving human participants complied with applicable national regulations and ethical standards. The study protocol was approved by the Independent Bioethical Commission for Issues of Scientific Research at the University of Gdańsk (Resolution No. NKBBN/144/2021). The study was carried out in accordance with the principles set out in the Declaration of Helsinki.

The first section of the questionnaire collected sociodemographic information. The specific items and response options are presented in Table 1. The second section included items regarding PA and was assessed using four-point response scales. Responses comprised four agreement categories (“definitely agree”, “mostly agree”, “rather disagree”, and “definitely disagree”) and a separate “not applicable” option. Three outdoor activity-related behaviors were analyzed: walking in public places, such as parks, beaches, or boulevards; walking in the neighborhood; and outdoor sports activity, such as running or cycling (Table 2). The final section consisted of ten items based on the Perceived Stress Scale (PSS), administered in its standard time-frame version. Items 4, 7, and 8 were reverse scored. Item 5 was positively worded in Polish (“things were going my way”) and was therefore reverse-scored, whereas its Italian counterpart was phrased negatively (“things were not going as I would have liked”) and was scored in its original direction. Consequently, higher scores consistently indicated greater perceived stress in both datasets. PSS totals were calculated by summing the scored items. Validated Polish and Italian translations of the scale were used.

Because the PSS variable did not meet the assumption of normal distribution, non-parametric methods were used for the principal group comparisons. Responses were initially analyzed across the original response categories and were additionally dichotomized into agreement (“definitely agree” and “mostly agree”) and disagreement groups (“rather disagree” and “definitely disagree”) for selected comparisons. This dichotomization was used to provide a directly interpretable contrast between endorsement and non-endorsement of each broadly phrased activity statement. “Not applicable” remained a separate substantive response and, together with missing responses, was excluded from the corresponding dichotomized analysis.

Survey data were collected online via Google Forms and then exported to Microsoft Excel (Microsoft Corporation, Redmont, WA, USA) spreadsheets. Statistical analyses were conducted using STATISTICA version 10.0 (StatSoft Inc., Tulsa, OK, USA). Because the PSS score was not normally distributed, the Kruskal–Wallis test was used for comparisons across multiple response categories and the Mann-Whitney U test for dichotomous comparisons. A multivariable model was constructed to evaluate the association between the PA variables and the odds of belonging to the high-stress category while adjusting for relevant covariates.

The logistic regression outcome was membership in an operationally defined high perceived stress category (PSS-10 ≥ 27), consistent with categorizations used in previous studies [24,25,26]. Because the PSS-10 is not a diagnostic instrument and has no formally established diagnostic cut-offs, this threshold was used only to define an analytical group. The multivariable model included variables previously identified as significantly associated with perceived stress in the parent analysis [4], including country, self-rated health status, employment status, age, sex, education, marital status, work-from-home status, self-assessed household financial situation, and city size. Results were reported as odds ratios (ORs) with 95% confidence intervals.

A *p*-value below 0.05 was considered statistically significant. Effect sizes for categorical comparisons between the Polish and Italian subsamples were quantified using Cramer’s V. As a sensitivity analysis addressing the unequal national sample sizes, the adjusted logistic regression model was refitted using normalized inverse country-sample-size weights, so that the Polish and Italian subsamples each contributed 50% of the total analytical weight. The effect-size and weighting analyses were refitted in Python version 3.12.13 using pandas version 2.2.3, NumPy version 2.3.5, and SciPy version 1.17.0.

Figures were created in Microsoft Excel (Microsoft Corporation, Redmont, WA, USA). The authors used ChatGPT 5.5 and ChatGPT 5.6 Sol (OpenAI, San Francisco, CA, USA) for language editing, structural refinement of the manuscript, preliminary assistance in identifying relevant literature and preparation of the Python code for the effect-size and country-balanced sensitivity analysis. All outputs were critically evaluated and verified by the authors. The authors remain fully responsible for the final content of the manuscript.

## 3. Results

The main sociodemographic characteristics of the Polish and Italian respondents are summarized in Table 1. A more detailed description of the source population survey has been published previously [4]. The two national subsamples differed across several variables relevant to perceived stress and outdoor activity. Compared with the Italian subsample, the Polish subsample included a higher proportion of women (76.2% vs. 62.2%) and younger respondents, whereas the Italian subsample included relatively more respondents aged 45 years or older. Country differences were also observed for place of residence, marital status, employment status, household financial situation, and self-rated health. The largest differences were observed for place of residence and age, followed by employment status. These differences support the inclusion of country as a core covariate in the multivariable model.

The Polish and Italian subsamples differed significantly in the distribution of responses for all three outdoor activity variables (Table 2). These findings indicate that the two national subsamples differed not only in sociodemographic profile and perceived stress, but also in the outdoor activity patterns analyzed in this study.

### 3.1. Walking in Public Places and Perceived Stress

Perceived stress differed significantly according to the reported frequency of walking in public places (Kruskal–Wallis H = 14.74; *p* = 0.005). Participants who agreed that they went for walks in public places had lower mean PSS scores than those who disagreed (Table 3). Also in the dichotomized analysis, respondents who reported walking in public places had a lower mean PSS score than those who did not (20.40 vs. 21.61; *p* < 0.001) (Table 4).

### 3.2. Outdoor Sports Activity and Perceived Stress

A significant association was also observed between outdoor sports activity and perceived stress (Kruskal–Wallis H = 20.67; *p* < 0.001). Participants who reported engaging in outdoor sports (e.g., running, cycling) had lower mean PSS scores than those who did not (Table 5). The difference was also significant in the dichotomized analysis (20.59 vs. 21.70, *p* < 0.001) (Table 6).

### 3.3. Walking in the Neighborhood and Perceived Stress

Perceived stress differed across the original categories of neighborhood walking (Kruskal–Wallis H = 19.89; *p* < 0.001) (Table 7). In the dichotomized analysis, the agreement group had a slightly higher mean PSS score than the disagreement group (21.76 vs. 21.26, *p* = 0.018) (Table 8). Therefore, the association between neighborhood walking and perceived stress seems less consistent than that observed for public-place walking or outdoor sports activity.

### 3.4. Multivariable Analysis

**Figure 1:**
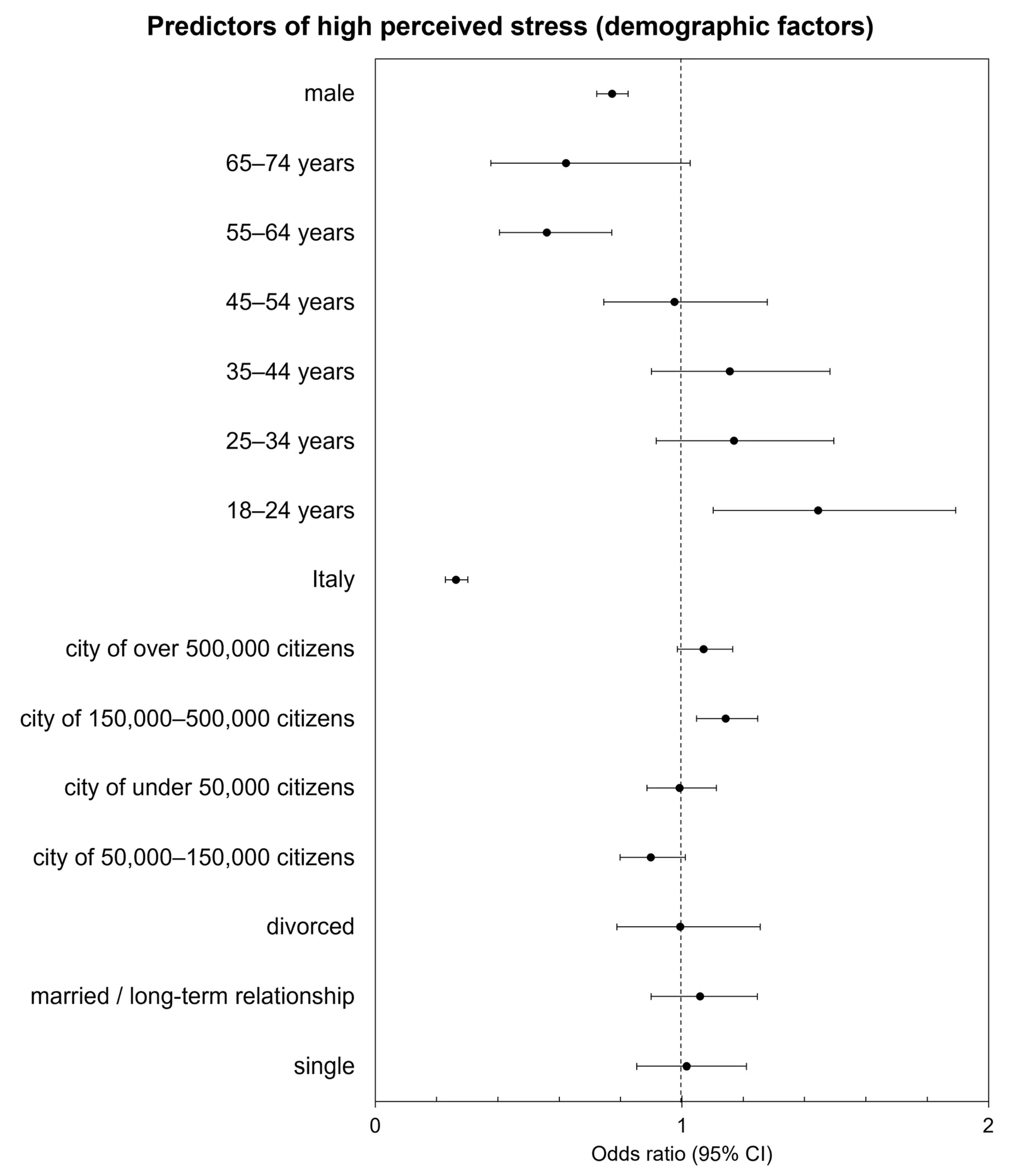
Demographic predictors of high perceived stress. The dashed vertical line indicates OR = 1.00.

In the demographic and country-related panel, male sex was associated with lower odds of belonging to the high-stress category (OR = 0.772, 95% CI: 0.722–0.825, *p* < 0.001). Compared with the reference age group, respondents aged 18–24 years had higher odds of high perceived stress (OR = 1.444, 95% CI: 1.102–1.893, *p* = 0.008), whereas those aged 55–64 years had lower odds (OR = 0.559, 95% CI: 0.405–0.771, *p* < 0.001). Respondents from Italy had markedly lower odds of high perceived stress than respondents from Poland (OR = 0.263, 95% CI: 0.229–0.302, *p* < 0.001). Among place-of-residence categories, living in a city with 150,000–500,000 inhabitants was associated with higher odds of high perceived stress (OR = 1.143, 95% CI: 1.048–1.247, *p* = 0.002). Marital status categories were not independently associated with the outcome. The results are presented in Figure 1. The full adjusted multivariable logistic regression model, including all covariates entered into the analysis, is presented in Appendix A.

**Figure 2:**
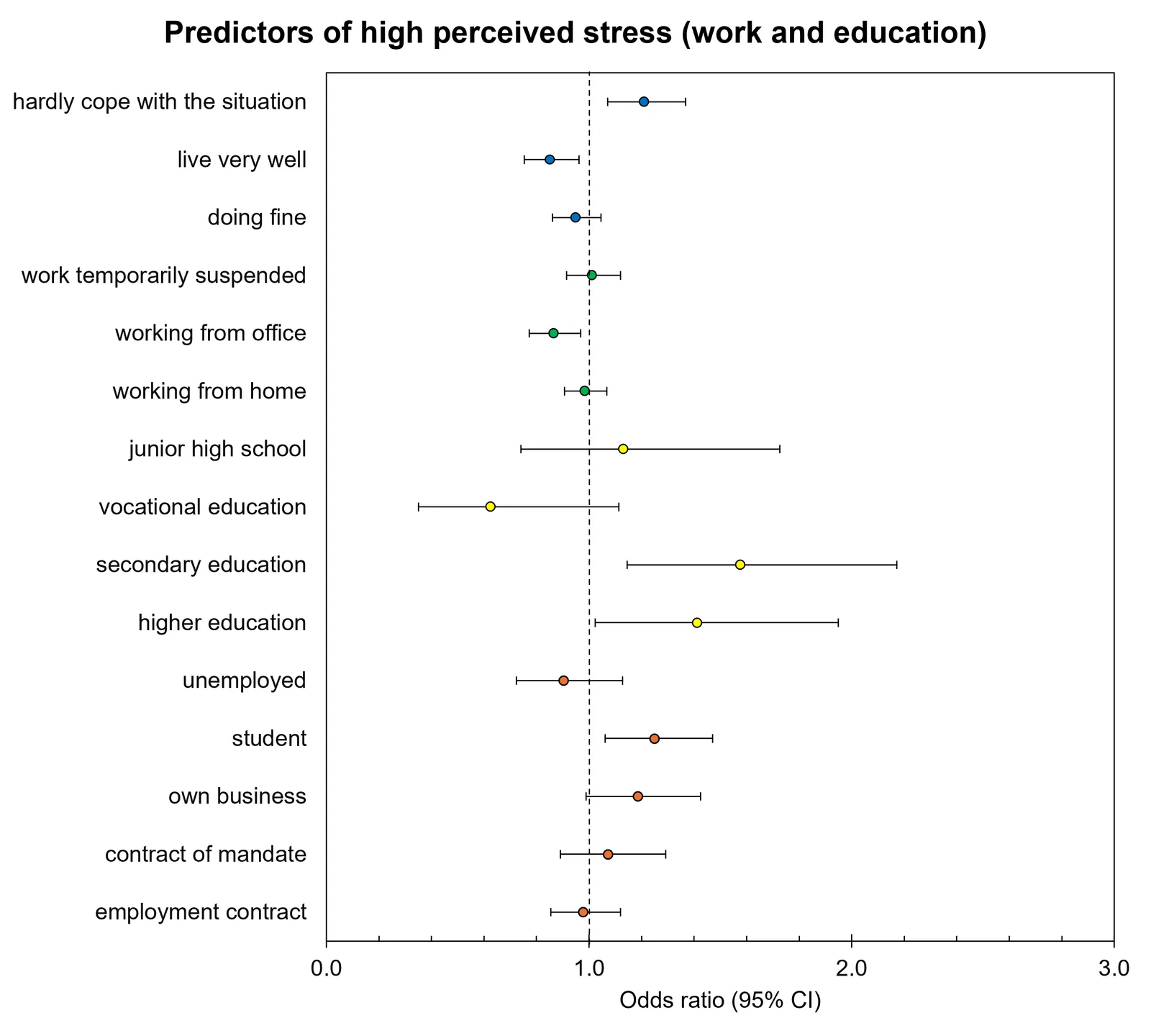
Work- and education-related predictors of high perceived stress. The dashed vertical line indicates OR = 1.00.

The second forest plot summarizes work-, education-, and financial-status-related predictors. Student status was associated with higher odds of high perceived stress (OR = 1.249, 95% CI: 1.061–1.471, *p* = 0.008), while working from the office was associated with lower odds compared with the reference category (OR = 0.866, 95% CI: 0.773–0.968, *p* = 0.012). Higher and secondary education were also associated with higher odds of high perceived stress (OR = 1.412, 95% CI: 1.024–1.949, *p* = 0.035 and OR = 1.577, 95% CI: 1.145–2.171, *p* = 0.005, respectively). Household financial situation was independently associated with the outcome: respondents reporting that they lived very well had lower odds of high perceived stress (OR = 0.851, 95% CI: 0.753–0.961, *p* = 0.010), whereas those who reported hardly coping with the situation had higher odds (OR = 1.209, 95% CI: 1.070–1.368, *p* = 0.002). The results are presented in Figure 2.

**Figure 3:**
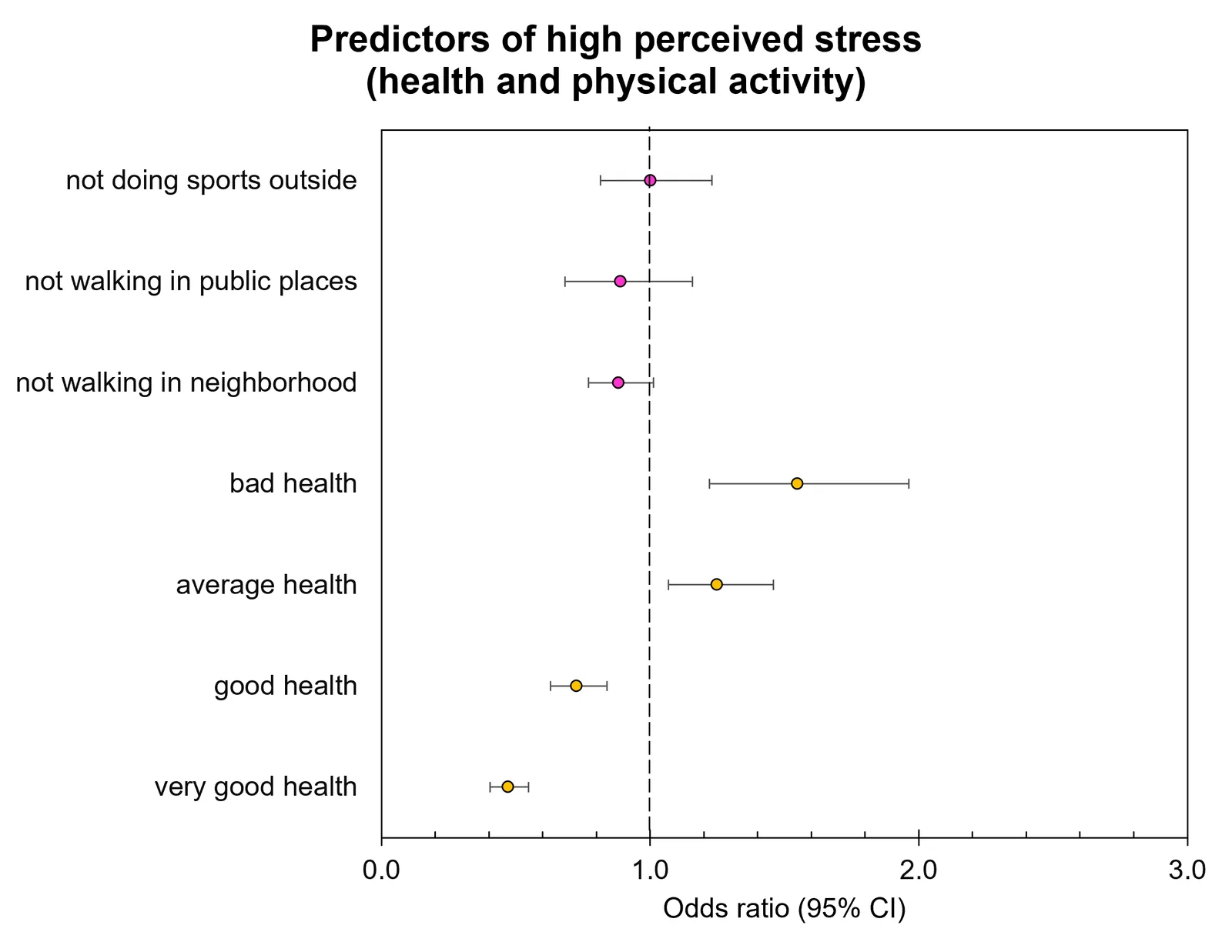
Health status and outdoor physical activity predictors of high perceived stress. The dashed vertical line indicates OR = 1.00.

The third forest plot presents health status and outdoor physical activity variables. Self-rated health status was strongly associated with high perceived stress. Very good and good health were associated with lower odds (OR = 0.471, 95% CI: 0.404–0.548, *p* < 0.001 and OR = 0.726, 95% CI: 0.629–0.839, *p* < 0.001, respectively), whereas average and bad health were associated with higher odds compared with the reference category (OR = 1.248, 95% CI: 1.069–1.458, *p* = 0.005 and OR = 1.548, 95% CI: 1.221–1.963, *p* < 0.001, respectively). None of the three activity variables showed an independent association with high perceived stress: not walking in public places (OR = 0.889, 95% CI: 0.683–1.158, *p* = 0.384), not walking in the neighborhood (OR = 0.883, 95% CI: 0.771–1.013, *p* = 0.075), and not doing outdoor sports (OR = 1.002, 95% CI: 0.816–1.230, *p* = 0.983). The results are presented in Figure 3.

The conclusions remained unchanged in the country-balanced weighted sensitivity analysis. None of the three outdoor activity variables was associated with high perceived stress after assigning equal total analytical weight to the Polish and Italian samples (Appendix A).

## 4. Discussion

The present analysis showed that selected forms of outdoor activity were associated with perceived stress during the first wave of the COVID-19 pandemic. Participants who reported walking in public places and those who engaged in outdoor sports had lower PSS scores in unadjusted analyses. However, after adjustment for a broad range of sociodemographic and health-related variables, none of the three activity measures were independently associated with high perceived stress. The findings of the present study are in line with evidence from pandemic-era research indicating that PA may have played a protective role for mental health during periods of lockdown and stay-at-home restrictions. Studies conducted during the first year of the COVID-19 pandemic showed that higher levels of PA were generally associated with fewer negative mental health symptoms, including depression, anxiety, fear, and poorer well-being, across different age groups [14,17,18,19,20,21,27].

Previous pandemic-era studies have often conceptualized PA primarily in quantitative terms, focusing on the amount, intensity, frequency, duration, or metabolic equivalent of task (MET) rather than on the specific behavioral context in which activity was performed [17,19,27]. For example, studies using IPAQ-based indicators examined walking, moderate and vigorous PA, and total MET-minutes per week, showing that higher activity volume was generally associated with better mental health outcomes during confinement [17]. Other studies considered time spent outdoors or broad PA categories but did not distinguish between specific outdoor activities [21]. In contrast, studies that did examine specific activity types focused mainly on home-based or indoor forms of exercise, including stretching, resistance training, household chores, family games, rope skipping, badminton, Chinese traditional sports, yoga, video dancing, sensory-motor games, and whole-body vibration [18,20].

Our findings add to this literature by suggesting that the behavioral context of outdoor activity may be important. In the present study, walking in public places and outdoor sports were associated with lower mean PSS scores, while neighborhood walking was associated with a slightly higher mean score. These differences were small and did not persist as independent associations in the adjusted model. They should therefore not be interpreted as evidence that one form of outdoor activity protected against stress more effectively than another.

Evidence from previous studies suggests that PA should not be treated as a homogeneous exposure, as different types of activity may have different associations with mental health. In a large cross-sectional study of over 1.2 million adults in the United States (U.S.), Chekroud et al. showed that exercise was associated with fewer days of poor mental health, but the magnitude of this association differed by exercise type, duration, and frequency. Notably, the strongest associations were observed for team sports, cycling, and aerobic/gym activities, whereas walking was also beneficial but showed a weaker association than several more structured or vigorous forms of exercise [28]. Similarly, Bafageeh and Loux, using data from a large U.S. adult sample, found that participation in different types of PA was associated with lower odds of mental health problems, with running, sports, and weightlifting showing the strongest relationships, while household activities showed the weakest associations [22]. Outdoor sports may reflect a more intentional and structured coping behavior than walking. During lockdown conditions, regular outdoor sports may have helped individuals preserve routine, goal-directed behavior, and a sense of control, all of which could be relevant for perceived stress [29,30,31]. In contrast, walking in the neighborhood may have represented a more necessity-driven form of movement, not necessarily undertaken as deliberate exercise or recreation.

The higher risk of high perceived stress observed among younger respondents and students is consistent with previous research indicating that young adults and university students experienced substantial psychological burden during the COVID-19 pandemic [20,29,30,31,32,33]. Disruption of education, transition to online learning, reduced peer interaction, uncertainty about the future, and loss of daily structure may have contributed to this vulnerability. These findings suggest that interventions promoting safe and regular PA during periods of restriction may be particularly important for younger populations and students.

The ability to maintain outdoor sports during lockdown may also have depended on contextual factors not fully captured in the present analysis. During the first pandemic wave, restrictions did not affect all branches of the economy in the same way. In both Poland and Italy, sectors such as hospitality, tourism, culture, sports and fitness facilities, and many personal services were suspended or strongly limited, whereas healthcare, food supply, logistics, public utilities, and selected essential retail activities continued to operate [34,35]. These differences may have influenced daily routines, time availability, perceived safety, financial uncertainty, and opportunities to maintain regular outdoor exercise.

Country of residence was strongly associated with perceived stress, consistent with the parent analysis in which Polish respondents had higher PSS scores than Italian respondents and the countries differed in compliance and activity-related patterns [4].

The results have practical implications for public health planning during future epidemic waves or other periods of social restriction. Although mobility restrictions may be necessary to reduce infection transmission, they can also limit opportunities for PA, increase sedentary behavior, and reduce access to outdoor spaces that support mental well-being. Therefore, where epidemiologically feasible, public health strategies should aim to preserve safe opportunities for outdoor PA, for example by maintaining access to parks, green spaces, and low-crowded outdoor recreational areas while implementing appropriate distancing measures. This is consistent with recommendations emphasizing that parks and green spaces may support both physical and mental health during pandemics, if access is managed safely [36,37,38]. Public health messaging should also highlight the potential mental health value of maintaining regular, structured exercise routines outdoors [12]. In the context of our findings, such recommendations may be especially relevant for outdoor sports.

A strength of this study is the large binational sample and the ability to adjust for numerous covariates previously associated with perceived stress. The inclusion of respondents from Poland and Italy is informative because the two subsamples differed in stress profile and sociodemographic composition. The interaction analyses suggest that a country-adjusted pooled model is a reasonable analytical approach. Another advantage of the study is that the distinction between different forms of outdoor activity also provides a more nuanced perspective than analyses based solely on total PA.

Several limitations should be acknowledged. First, the cross-sectional design of the study precludes causal inference. Second, PA was assessed using self-reported declarations, which may be affected by recall bias and subjective interpretation of activity categories. Moreover, the available data did not include detailed information on the frequency, duration, intensity, or regularity of specific activities, limiting the ability to assess dose–response relationships. Third, the Polish sample was substantially larger than the Italian sample and the national subsamples differed sociodemographically, limiting cross-country comparability and the precision of interaction estimates. Moreover, the dichotomization of the PSS-10 score at ≥27 resulted in a loss of information regarding variation across the full range of perceived stress and may have reduced statistical power. Consequently, the findings should be interpreted as associations with the odds of belonging to the operationally defined high-stress group, rather than associations across the full continuum of perceived stress or evidence of clinically diagnosed stress. Finally, although the multivariable model adjusted for several sociodemographic and health-related factors, residual confounding cannot be excluded.

## 5. Conclusions

In this secondary cross-sectional analysis, self-reported walking in public places and outdoor sports were associated with lower PSS scores in unadjusted comparisons, whereas neighborhood walking showed a small difference in the opposite direction. After correcting the dichotomized activity variables and adjustment for country, sociodemographic characteristics, health, and work-related factors, none of the three activity measures were independently associated with high perceived stress. The findings therefore do not support a robust activity-specific protective association. Future longitudinal studies should use validated measures of activity frequency, duration, and intensity and should recruit more balanced national samples.

## Figures and Tables

**Table 1:** Characteristics of the study population.

Variable	Category	Total (*N* = 7113), *n* (%)	Polish (*N* = 6169), *n* (%)	Italian (*N* = 944), *n* (%)	*V*	*p*
sex	female	5285 (74.3)	4698 (76.2)	587 (62.2)	0.111	<0.001
	male	1749 (24.6)	1402 (22.7)	347 (36.8)		
	NOS	79 (1.1)	69 (1.1)	10 (1.1)		
age	18–24	2036 (28.6)	1945 (31.5)	91 (9.6)	0.279	<0.001
	25–34	2277 (32.0)	2034 (33.0)	243 (25.7)		
	35–44	1473 (20.7)	1270 (20.6)	203 (21.5)		
	45–54	736 (10.3)	552 (8.9)	184 (19.5)		
	55–64	439 (6.2)	259 (4.2)	180 (19.1)		
	65+	147 (2.1)	109 (1.8)	38 (4.0)		
place of residence	village/town	909 (12.8)	692 (11.2)	217 (23.0)	0.369	<0.001
	city with <50,000 inhabitants	880 (12.4)	730 (11.8)	150 (15.9)		
	city 50,000–150,000 inhabitants	989 (13.9)	604 (9.8)	385 (40.8)		
	city 150,000–500,000 inhabitants	1990 (28.0)	1894 (30.7)	96 (10.2)		
	city with >500,000 inhabitants	2341 (32.9)	2249 (36.5)	92 (9.7)		
marital status	single	2956 (41.6)	2690 (43.6)	266 (28.2)	0.107	<0.001
	married/relationship	3779 (53.1)	3166 (51.3)	613 (64.9)		
	divorced/separated	304 (4.3)	255 (4.1)	49 (5.2)		
	widow/widower	73 (1.0)	58 (0.9)	15 (1.6)		
educational level	lower secondary or lower	123 (1.7)	89 (1.4)	34 (3.6)	0.069	<0.001
	secondary school	2111 (29.7)	1821 (29.5)	290 (30.7)		
	higher education	4793 (67.4)	4194 (68.0)	599 (63.5)		
	professional qualification	86 (1.2)	65 (1.1)	21 (2.2)		
occupational status	employed (with contract)	3474 (48.8)	3002 (48.7)	472 (50.0)	0.150	<0.001
	entrepreneur/self-employed	596 (8.4)	481 (7.8)	115 (12.2)		
	freelance/civil-law contract	386 (5.4)	375 (6.1)	11 (1.2)		
	worker without contract	16 (0.2)	0 (0.0)	16 (1.7)		
	student	1917 (27.0)	1777 (28.8)	140 (14.8)		
	unemployed	305 (4.3)	242 (3.9)	63 (6.7)		
	pensioner	202 (2.8)	152 (2.5)	50 (5.3)		
	other	217 (3.1)	140 (2.3)	77 (8.2)		
work conditions	business suspended	1083 (15.2)	932 (15.1)	151 (16.0)	0.033	0.049
	smart working	3006 (42.3)	2582 (41.9)	424 (44.9)		
	business as usual	1074 (15.1)	931 (15.1)	143 (15.1)		
	not applicable	1947 (27.4)	1724 (27.9)	223 (23.6)		
current financial condition	good	1660 (23.3)	1543 (25.0)	117 (12.4)	0.111	<0.001
	fairly good	4188 (58.9)	3589 (58.2)	599 (63.5)		
	struggling to cope	879 (12.4)	716 (11.6)	163 (17.3)		
	cannot cope	153 (2.2)	134 (2.2)	19 (2.0)		
	not answer	231 (3.2)	187 (3.0)	44 (4.7)		
self-perceived health status	very fragile	24 (0.3)	17 (0.3)	7 (0.7)	0.101	<0.001
	fragile	121 (1.7)	87 (1.4)	34 (3.6)		
	average	879 (12.4)	719 (11.7)	160 (16.9)		
	good	3382 (47.5)	2911 (47.2)	471 (49.9)		
	very good	2706 (38.0)	2435 (39.5)	271 (28.7)		

*N*—number, V—Cramer’s V, *p*—statistical significance, NOS—not otherwise specified.

**Table 2:** Outdoor activity patterns by country according to original response categories.

Outdoor Activity	Response	Poland (*N* = 6169), *n* (%)	Italy (*N* = 944), *n* (%)	*V*	*p*
Walking in public places	definitely agree	144 (2.3)	66 (7.0)	0.252	<0.001
	mostly agree	235 (3.8)	133 (14.1)		
	rather disagree	781 (12.7)	143 (15.1)		
	definitely disagree	4397 (71.3)	387 (41.0)		
	not applicable	612 (9.9)	209 (22.1)		
	missing	0 (0.0)	6 (0.6)		
Walking in the neighborhood	definitely agree	1438 (23.3)	276 (29.2)	0.156	<0.001
	mostly agree	1379 (22.4)	257 (27.2)		
	rather disagree	1288 (20.9)	104 (11.0)		
	definitely disagree	1855 (30.1)	205 (21.7)		
	not applicable	209 (3.4)	98 (10.4)		
	missing	0 (0.0)	4 (0.4)		
Outdoor sports activity	definitely agree	365 (5.9)	124 (13.1)	0.19	<0.001
	mostly agree	382 (6.2)	129 (13.7)		
	rather disagree	656 (10.6)	124 (13.1)		
	definitely disagree	3825 (62.0)	333 (35.3)		
	not applicable	941 (15.3)	225 (23.8)		
	missing	0 (0.0)	9 (1.0)		

*N*—number, V—Cramer’s V, *p*—statistical significance.

**Table 3:** PSS scores according to going out for walks to public places.

“I Go Out for a Walk to Public Places (Parks, Beaches, Boulevard, etc.)”	*N*	Mean PSS	SD
not applicable	821	21.24	8.35
definitely agree	210	20.22	8.36
mostly agree	368	20.50	7.81
rather disagree	924	21.63	7.03
definitely disagree	4784	21.60	7.66
**Total**	**7107**	**21.47**	**7.70**

*N*—number, PSS—Perceived Stress Scale, SD—standard deviation.

**Table 4:** PSS scores according to going out for walks to public places—dichotomized analysis.

“I Go Out for a Walk to Public Places (Parks, Beaches, Boulevard, etc.)”	*N*	Mean PSS	SD
agree (definitely agree & mostly agree)	578	20.40	8.01
disagree (definitely disagree & rather disagree)	5708	21.61	7.56

*N*—number, PSS—Perceived Stress Scale, SD—standard deviation.

**Table 5:** PSS scores according to doing sports outside.

“I Do Sports Outside, e.g., Running, Cycling, etc.”	*N*	Mean PSS	SD
not applicable	1166	21.27	7.86
definitely agree	489	20.43	8.30
mostly agree	511	20.75	7.55
rather disagree	780	21.61	7.17
definitely disagree	4158	21.72	7.67
**Total**	**7104**	**21.48**	**7.69**

*N*—number, PSS—Perceived Stress Scale, SD—standard deviation.

**Table 6:** PSS scores according to doing sports outside—dichotomized analysis.

“I Do Sports Outside, e.g., Running, Cycling, etc.”	*N*	Mean PSS	SD
agree (definitely agree & mostly agree)	1000	20.59	7.92
disagree (definitely disagree & rather disagree)	4938	21.70	7.59

*N*—number, PSS—Perceived Stress Scale, SD—standard deviation.

**Table 7:** PSS scores according to going out for walks in the neighborhood.

“I Go Out for a Walk in My Neighborhood”	*N*	Mean PSS	SD
not applicable	307	20.52	8.77
definitely agree	1714	21.50	7.82
mostly agree	1636	22.03	7.25
rather disagree	1392	21.75	7.21
definitely disagree	2060	20.94	8.05
**Total**	**7109**	**21.47**	**7.70**

*N*—number, PSS—Perceived Stress Scale, SD—standard deviation.

**Table 8:** PSS scores according to going out for walks in the neighborhood—dichotomized analysis.

“I Go Out for a Walk in My Neighborhood”	*N*	Mean PSS	SD
agree (definitely agree & mostly agree)	3350	21.76	7.55
disagree (definitely disagree & rather disagree)	3452	21.26	7.73

*N*—number, PSS—Perceived Stress Scale, SD—standard deviation.

## Data Availability

The data presented in this study is available on request from the corresponding author.

## Abbreviations

The following abbreviations are used in this manuscript:

COVID-19	coronavirus disease 2019
PA	physical activity
PSS	Perceived Stress Scale
OR	odds ratio
CI	confidence interval
N	sample size
SD	standard deviation
*p*	*p*-value (statistical significance)
V	Cramer’s V
NOS	not otherwise specified
MET	metabolic equivalent of task
IPAQ	International Physical Activity Questionnaire
U.S.	United States

## Appendix A

**Table A1:** Multivariable model of factors associated with belonging to the high-stress category.

Predictor	Category	OR	95% CI	*p*
sex	male	0.772	0.722–0.825	<0.001
age	18–24 years	1.444	1.102–1.893	0.008
	25–34 years	1.170	0.916–1.495	0.210
	35–44 years	1.156	0.901–1.483	0.254
	45–54 years	0.976	0.745–1.278	0.859
	55–64 years	0.559	0.405–0.771	<0.001
	65–74 years	0.622	0.377–1.027	0.064
country	Italy	0.263	0.229–0.302	<0.001
city size	50,000–150,000 citizens	0.899	0.798–1.011	0.077
	under 50,000 citizens	0.993	0.886–1.112	0.898
	150,000–500,000 citizens	1.143	1.048–1.247	0.002
	over 500,000 citizens	1.071	0.985–1.166	0.110
marital status	single	1.016	0.853–1.211	0.858
	married/long-term relationship	1.059	0.900–1.246	0.492
	divorced	0.995	0.788–1.255	0.965
employment status	employment contract	0.978	0.854–1.119	0.742
	contract of mandate	1.073	0.891–1.292	0.459
	own business	1.186	0.988–1.425	0.067
	student	1.249	1.061–1.471	0.008
	unemployed	0.904	0.724–1.128	0.370
work from home	working from home	0.984	0.906–1.068	0.697
	working from office	0.866	0.773–0.968	0.012
	work temporarily suspended	1.011	0.914–1.119	0.826
education	higher	1.412	1.024–1.949	0.035
	secondary	1.577	1.145–2.171	0.005
	vocational	0.625	0.351–1.113	0.110
	junior high school	1.131	0.741–1.726	0.568
household financial situation	doing fine	0.949	0.861–1.046	0.295
	live very well	0.851	0.753–0.961	0.010
	hardly cope with the situation	1.209	1.070–1.368	0.002
	refuse to answer	0.786	0.621–0.994	0.045
health status	very good	0.471	0.404–0.548	<0.001
	good	0.726	0.629–0.839	<0.001
	average	1.248	1.069–1.458	0.005
	bad	1.548	1.221–1.963	<0.001
physical activity outside	not walking in the neighborhood	0.883	0.771–1.013	0.075
	not walking in public places	0.889	0.683–1.158	0.384
	not doing sports outside	1.002	0.816–1.230	0.983

OR—odds ratio, CI—confidence interval, *p*—statistical significance.

**Table A2:** Comparison of the primary and country-balanced weighted logistic regression models.

Outdoor Activity Variable	Primary Model OR (95% CI)	*p*	Country-Balanced Model OR (95% CI)	*p*
not walking in public places	0.889 (0.683–1.158)	0.384	0.901 (0.616–1.317)	0.591
not walking in the neighborhood	0.883 (0.771–1.013)	0.075	1.022 (0.846–1.235)	0.821
not doing sports outside	1.002 (0.816–1.230)	0.983	1.106 (0.831–1.473)	0.489

OR—odds ratio; CI—confidence interval; *p*—statistical significance.
